# Challenges in developing therapies in fragile X syndrome: how the FXLEARN trial can guide research

**DOI:** 10.1172/JCI175036

**Published:** 2024-03-01

**Authors:** Jeffrey L. Neul

**Affiliations:** Vanderbilt Kennedy Center and Departments of Pediatrics, Pharmacology, and Special Education, Vanderbilt University Medical Center, Nashville, Tennessee, USA.

## Abstract

Fragile X syndrome (FXS), the most common inherited cause of intellectual disability and the single-gene cause of autism, is caused by decreased expression of the fragile X messenger ribonucleoprotein protein (FMRP), a ribosomal-associated RNA-binding protein involved in translational repression. Extensive preclinical work in several FXS animal models supported the therapeutic potential of decreasing metabotropic glutamate receptor (mGluR) signaling to correct translation of proteins related to synaptic plasticity; however, multiple clinical trials failed to show conclusive evidence of efficacy. In this issue of the *JCI*, Berry-Kravis and colleagues conducted the FXLEARN clinical trial to address experimental design concerns from previous trials. Unfortunately, despite treatment of young children with combined pharmacological and learning interventions for a prolonged period, no efficacy of blocking mGluR activity was observed. Future systematic evaluation of potential therapeutic approaches should evaluate consistency between human and animal pathophysiological mechanisms, utilize innovative clinical trial design from FXLEARN, and incorporate translatable biomarkers.

## Mouse models reveal pathophysiological mechanisms in fragile X syndrome

Neurodevelopmental disorders (NDDs) are a broad set of conditions manifesting due to nervous system dysfunction causing a range of clinical features, including intellectual disability (ID), communication dysfunction, behavioral and emotional problems, and motor impairments. Cumulatively, NDDs are highly prevalent (1) and affect the quality of life of affected individuals. Understanding causes of NDDs, including brain injury, infection, environmental exposures, social deprivation, and genetic causes, guides preventative strategies and interventional approaches to decrease the impact on affected individuals, families, and society (2). The determination of genetic causes of NDDs has enabled development of animal and cell models to identify pathophysiological mechanisms and develop therapies.

Fragile X syndrome (FXS) is an X-linked NDD that affects approximately 1 in 4,000 males and females (3) and is the most common inherited cause of ID and single-gene cause of autism spectrum disorder (ASD). While physical features and medical problems are present in FXS, the most impactful issues are learning difficulties associated with ID, problematic behaviors, and challenges with social interactions, with approximately 50% affected males and about 20% affected females meeting criteria for ASD (3). Current therapies have limited effectiveness treating behavioral issues and do not address cognitive problems (3), representing a substantial unmet need (4, 5).

Most cases of FXS are caused by CGG trinucleotide repeat expansion (more than 200 repeats) within the promoter of the fragile X messenger ribonucleoprotein 1 gene (*FMR1*) gene, leading to promoter hypermethylation, transcriptional silencing, and decreased expression of the fragile X messenger ribonucleoprotein protein (FMRP) (6). FMRP is a ribosomal-associated RNA-binding protein involved in translational repression (4). FMRP is found within neuronal dendrites and regulates activity-dependent synthesis of proteins related to synaptic plasticity, involving ERK-, PI3K-, and mTOR-dependent signaling pathways (4). Experiments with translational inhibitors highlight the importance of FMRP in translational regulation by increasing cerebral protein synthesis and rescuing memory deficits in mouse models of FXS, which lack FMRP (7).

Disruption of protein translation in FXS mouse models led to evaluation of the therapeutic potential of modulation of neurotransmitter receptor activity that regulates translation of proteins critical for synaptic plasticity, such as group 1 metabotropic glutamate receptor (mGluR1 and mGluR5) activity (4). Specifically, the mGluR theory proposed that a substantial component of FXS pathophysiology is increased group 1 mGluR–dependent protein synthesis, leading to abnormal synaptic plasticity, dendritic morphology, and behavioral changes. Group 1 mGluR stimulation–dependent protein synthesis is required for synaptic plasticity, and abnormally increased mGluR-dependent synaptic plasticity is seen in FXS mouse models (7). In support of this theory, genetic reduction of mGluR5 activity corrected synaptic and behavioral phenotypes in mouse and fly models of FXS (4). Subsequently, extensive preclinical pharmacological work in mouse and fly models of FXS demonstrated that treatment with mGluR5 negative allosteric modulators (NAMs) improved synaptic, dendrite morphological, and behavioral phenotypes, pointing to the therapeutic potential of mGluR5 NAMs for the treatment of FXS (4).

## Previous clinical evaluation of mGluR NAM treatment

The robust preclinical evidence obtained from work conducted by many investigators in multiple species led to clinical evaluations of mGluR NAMs in FXS. An initial study of an mGluR5 NAM showed improvements in an endophenotype, prepulse inhibition (8), leading to two phase 2a studies of two mGluR5 NAMs (AFQ056 and RO4917523) in adults with FXS. Both demonstrated safety, tolerability, and signals of efficacy (3, 9). Subsequently, three phase 2b studies of these compounds in adolescents and adults with FXS characterized efficacy of these mGluR5 NAMs on behavioral features (10, 11), all of which failed to show efficacy and had large placebo effects, although post hoc analyses showed evidence of target engagement (3).

Issues related to trial design and primary outcome measures limited the ability to conclusively discount potential efficacy of these compounds. The relatively short duration of pharmacological intervention (three months) limited the ability to detect meaningful change in a lifelong NDD, and fixed dosing schedules precluded individual treatment optimization. Additionally, the primary outcome measures were caregiver-reported assessments of behavioral features that had large placebo effects, and the studies lacked objective performance assessments of cognition or functional skills. Further, the studies did not evaluate the effect of treatment on younger children, who could have a greater potential for benefit due to increased neuroplasticity in children. Finally, the potential benefit of combination of pharmacological treatment with targeted learning interventions was not assessed.

## Rigorous evaluation of mGluR NAM treatment

In this issue of the *JCI*, Berry-Kravis and colleagues (12) address these concerns and conclusively evaluate mGluR NAM treatment in FXS. The authors designed the FXLEARN trial, a placebo-controlled, double-blind study of an mGluR NAM (AFQ056) in young children (three to six years old) with FXS that incorporated numerous innovative features. To mitigate placebo effects, a four-month-long placebo lead-in period was used. Participants were then randomized to drug or placebo with a two-month flexible dosing titration period to individual maximally targeted dose (MTD), followed by six-month treatment on the MTD combined with a targeted language intervention, the Parent-Implemented Language Intervention (PILI). The PILI intervention was delivered by caregivers trained through a standardized process and adapted during the trial to address COVID pandemic restrictions, with fidelity and dose of PILI intervention assessed systematically. Importantly, the primary outcome was an objective, performance-based assessment of communication (Weighted Communication Scale [WCS]), video-captured during structured assessment and centrally scored by blinded high-fidelity coders using standardized methods. Additional secondary efficacy assessments and biomarkers were included, and participants had the option to continue in an eight-month open-label extension (12).

Despite the innovative features incorporated to address previous trial concerns and high participant retention despite COVID pandemic–related challenges, AFQ056 treatment was not beneficial. At the end of the placebo-controlled period, no differences between the treatment groups were observed in the primary outcome measure (WCS change) or key secondary outcome measures. In fact, the placebo group showed improved WCS score change, whereas the AFQ056 group did not. Subgroup analysis revealed that, while participants with high baseline communication skills showed similar language improvement in both treatment groups, language improvement was only observed in the placebo group for participants with low baseline communication skills, despite similar fidelity and participation in the PILI intervention. Behavioral issues related to AFQ056 treatment may have contributed to these findings, as behavioral measures trended toward improvement in the placebo group, but not in the AFQ056 group (12).

## Conclusions

Ultimately, the lack of treatment effect observed in the FXLEARN trial, combined with previous negative trials, provides conclusive evidence that reduction of mGluR5 activity is not beneficial for the treatment of cognition and behavior in people with FXS. This conclusion is unexpected considering the extensive preclinical evidence supporting this approach obtained from multiple species (4) and raises issues regarding the predictive validity and translatability of animal models to people in FXS. A recognized limitation of the FXS mouse model is the relatively subtle behavioral abnormalities that show marked strain variability (6) compared with the consistent dendritic and synaptic abnormalities corrected by reduction of mGluR5 activity. These findings raise concerns that corrections of morphological and synaptic phenotypes might have limited ability to predict human efficacy.

Furthermore, evidence has been mounting that challenges the generalizability of the mGluR theory of FXS pathology across species. In rat models, increasing rather than decreasing mGluR5 activity within the amygdala improved behavioral phenotypes (13). In humans, protein synthesis in the brain and blood mononuclear cells is decreased, rather than increased as found in animal models (14, 15), and human PET studies found reduced cerebral mGluR5 expression (16). Finally, human induced pluripotent stem cell–derived (iPSC-derived) neurons and cerebral organoid studies revealed difference in responses to and a lack of benefit from mGluR5 NAM treatment (17, 18).

The concerns about translatability between animal models and humans in FXS may lead to the proposal that animal models should be abandoned entirely in favor of studies in human-derived tissues, such as iPSC-derived organoids. Although iPSC-based systems have rapidly advanced, limitations exist with regard to developmental immaturity, lack of complex neural circuitry and phenotypes, and predictive validity (19). Animal models continue to have distinct value and should not be abandoned. Instead, the importance of evaluating the consistency of mechanisms and treatment responses across species, including humans, is critical for gaining confidence in the likelihood of efficacy in human clinical trials. Additionally, biomarkers translatable across animal models and humans, such as neurophysiological features (20, 21), need to be developed, validated, and utilized in preclinical and clinical studies.

**Figure 1:**
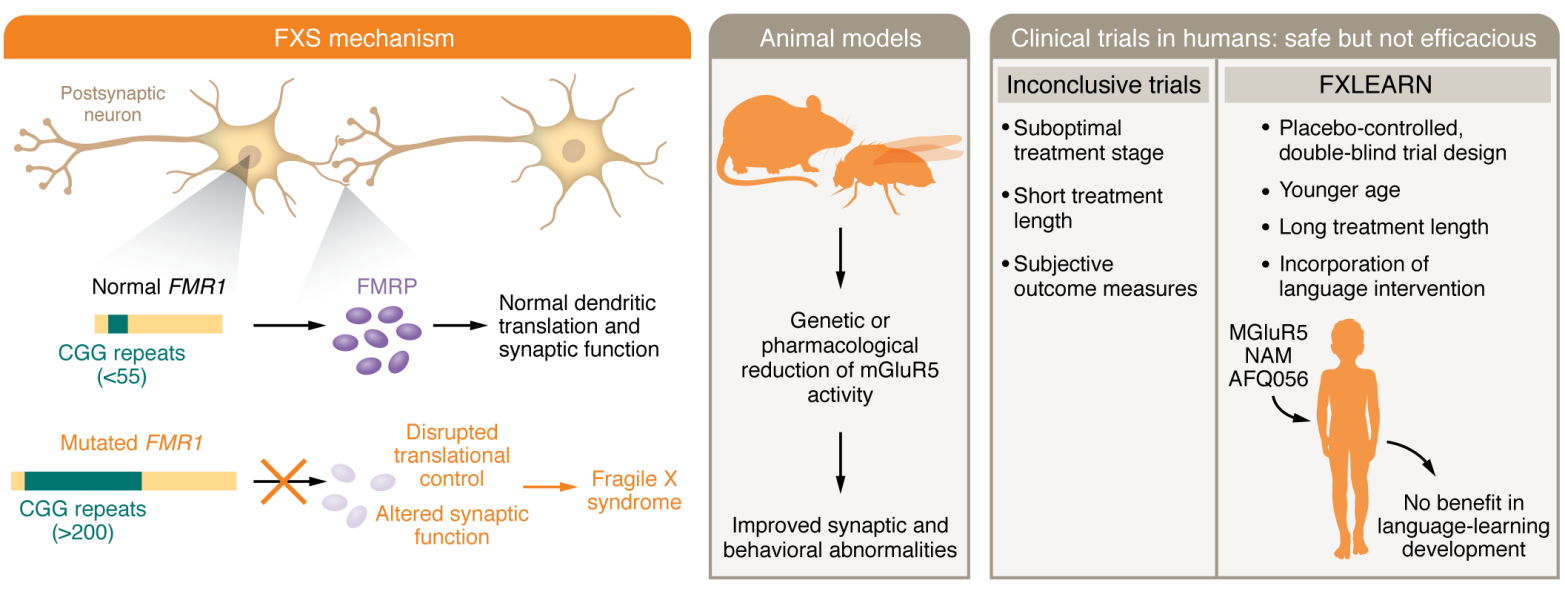
Development of clinical therapies in FXS requires mechanistic targets, translatable preclinical models, and rigorous trial design. Expansion of CGG trinucleotide repeats (more than 200) within the promoter of the *FMR1* gene results in promoter hypermethylation, transcriptional silencing, and decreased FMRP expression and causes FXS. FMRP is a ribosomal-associated RNA-binding protein that is involved in translational repression, is localized to neuronal dendrites, and regulates activity-dependent protein synthesis related to synaptic plasticity. Animal models suggest decreasing mGluR signaling might correct protein translation related to synaptic plasticity and improve phenotypes; however, multiple clinical trials have failed to show efficacy of this approach. The FXLEARN clinical trial (12) included an innovative study design, incorporating young children, combining pharmacological and learning interventions, and prolonging the treatment period. However, no efficacy of blocking mGluR activity was observed. Future studies should align human and animal pathophysiological mechanisms with rigorous clinical study design.

The failure of the predictions of the mGluR theory in FXS, supported by the most extensive work in any NDD, combined with the failure of other well-supported treatment approaches in FXS (22), might discourage further clinical development efforts in FXS and in NDDs. However, recent successful phase 3 trials in Rett syndrome (23) and CDKL5 deficiency disorder (24) argue against this nihilistic view. A number of additional therapeutic targets exist in FXS (3), and the innovative features of the FXLEARN trial (12) should be incorporated into future trials (Figure 1). Given progress in understanding of disease mechanisms and treatment targets in NDDs, clinical trials in these disorders should also utilize alternative trial design approaches, such as *n*-of-1 trials (25) and master protocol–based and adaptive-platform trials (26), to accelerate clinical therapy development for these prevalent and impactful conditions.

## Version 1 03/01/2024

Electronic publication
